# Post-polypectomy syndrome—a rare complication in colonoscopy procedures: a case report

**DOI:** 10.1093/jscr/rjac369

**Published:** 2022-08-30

**Authors:** Julián A Romo, Jorge David Peña, Laura A López, Carlos Figueroa, Horacio Garzon, Andrea Recamán

**Affiliations:** School of Medicine, Universidad del Rosario, Bogota D.C., Colombia; School of Medicine, Universidad del Rosario, Bogota D.C., Colombia; School of Medicine, Universidad del Rosario, Bogota D.C., Colombia; Department of Coloproctology, Hospital Universitario Mayor Méderi, Bogota D.C., Colombia; Department of Coloproctology, Hospital Universitario Mayor Méderi, Bogota D.C., Colombia; School of Medicine, Universidad del Rosario, Bogota D.C., Colombia

## Abstract

Post-polypectomy syndrome (PPS) is a complication that may arise after some colonoscopy procedures that require electrocoagulation, due to a transmural burn, which irritates the serous membrane. Its clinical presentation is similar to the one of intestinal perforation, but it has a favorable prognosis, and does not require surgical treatment. We report the case of a 55-year-old woman diagnosed with a polyp in the ascending colon, who was admitted for an endoscopic resection. After the procedure, she complained of nausea, emesis and abdominal pain in the right iliac fossa. She was transferred to the emergency department. An abdominal tomography showed cecal wall thickening without pneumoperitoneum. Therefore, the diagnosis of PPS was made and was managed with bowel rest, parenteral fluids and antibiotics, with full recovery. Despite of its low incidence, it is important to suspect this syndrome to avoid unnecessary surgical treatment and initiate medical management right away.

## INTRODUCTION

In the last decade colorectal cancer (CRC), cases have increased due to better-quality screening approach. A polyp found in any screening test can progress to CRC in 70–80% of the cases; they are generally resected with colonoscopy polypectomy [1]. Likewise, early stage CRC treated with methods such as endoscopic submucosal dissection (ESD) has a lower morbidity and mortality. Despite these benefits, and being relatively safe, endoscopic procedures carried out risks such as perforation and bleeding [1–3].

Post-polypectomy syndrome (PPS) is an endoscopic complications described for the first time by Waye in 1981, as a peritoneal irritation due to a transmural burn caused by electrocoagulation during endoscopic polypectomy. Its incidence is low, presenting in ~0.03–1.2% polypectomies [4, 5]. Given the rise of resections of large premalignant and malignant lesions through colonoscopy in the last decades, the recognition of PPS is essential, since early diagnosis and treatment are keys to avoid further complications.

## CASE REPORT

**Figure 1:**
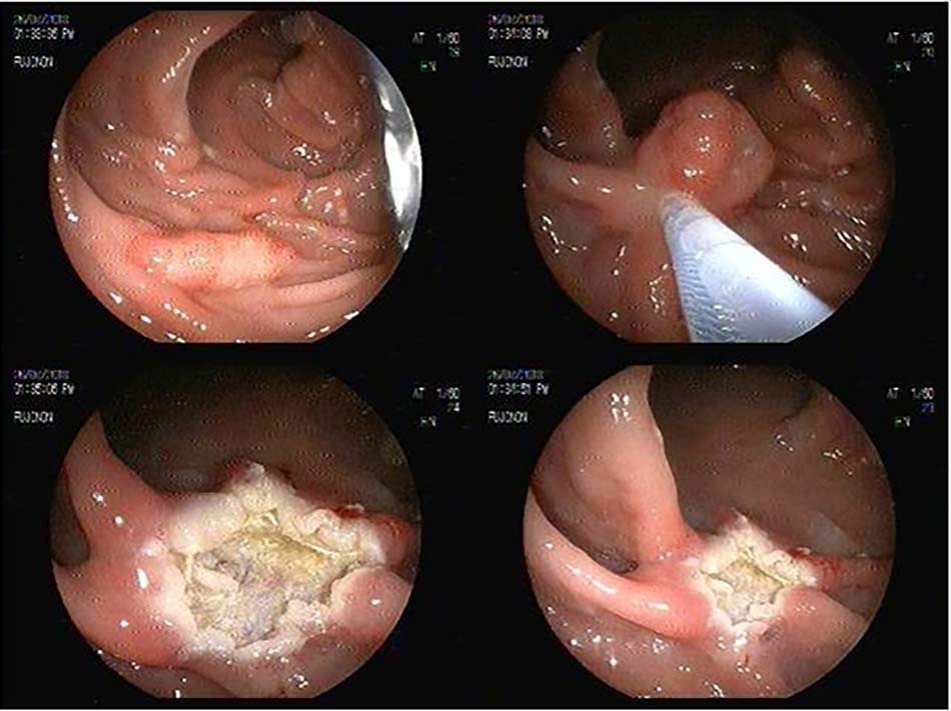
Endoscopic sessile polyp resection sequence with use of heated wire loop.

We report the case of a 55-year-old woman diagnosed with of a 3 cm × 4-cm polyp in the ascending colon, who was admitted for an endoscopic resection. At the beginning of the procedure the submucosa was infiltrated with adrenaline, methylene blue and saline solution, and then a heated wire loop resection was made (Fig. 1). Six hours after the procedure, the patient complained of nausea, multiple emetic episodes and abdominal pain in the right iliac fossa 10/10 intensity on an analogue scale of pain. She was transferred to the emergency department, her vital signs were normal and no physical examination signs of peritoneal irritation were found.

**Figure 2:**
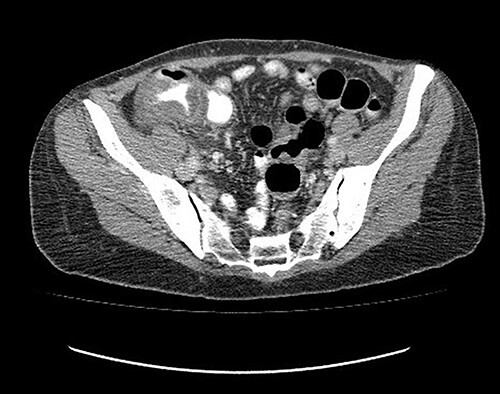
Abdominal tomography without pneumoperitoneum and with thickening of the concentric intestinal wall, without signs of intestinal perforation.

Analgesic management was prescribed, blood chemistry and thorax and abdomen X-ray were performed without abnormal results. An abdominal CT was performed, which showed concentric, focal thickening of the cecal walls, with a maximum thickness of 30 mm in the lateral wall, with enhancement with contrast, without evidence of pneumoperitoneum (Fig. 2). Given the history of recent polypectomy, patient’s symptoms, and the absence of signs of intestinal perforation, the diagnosis of PPS was made. Bowel rest, parenteral fluids and antibiotics (third-generation cephalosporin) were indicated, with a full recovery. The patient was discharged 24 h after admission. She was later evaluated in the coloproctology outpatient clinic, with complete resolution of symptoms. The histopathological study of the resected polyp was described as a hairy tubular adenoma. She was scheduled for a 1-year follow-up consult with a new colonoscopy study.

## DISCUSSION

PPS refers to the development of abdominal pain, fever and signs of peritoneal irritation in the absence of documented bowel perforation, after colonoscopy procedures that required electrocoagulation [3, 4]. The electric current applied to the mucosa towards the muscularis propria and serous membrane can lead to a transmural burn without perforation [4]. It presents with a sudden, high intensity abdominal pain, after 6–2 h after the intervention, peritoneal irritation, emetic episodes after the endoscopic procedure [4], and blood tests reveal inflammatory markers elevation (C-reactive protein, leukocytosis and neutrophilia; [4, 6]).

PPS can appear in 0.5–1.2% of patients taken to endoscopic polypectomy [3, 5, 7]. Some of the risk factors include: location in the ascending colon and the cecum, hypertension and polyp size greater than 40 mm [1, 5, 7]. The mechanism by which high blood pressure may contribute to PPS is unknown, it is thought to be related to endothelial dysfunction and atherosclerosis [4]. As to the distribution in the right colon, it is considered that its wall is thinner compared with the rest of the colon, this has been validated by cases of cecal perforation due to barotrauma during colonoscopy [4, 8]. PPS becomes more important in the scenario of an ESD; a procedure that has increased given the early detection of CRC; with ESD proctologist can reach ≥20-mm target lesion in which in block resection is more difficult [6]. Independent risk factors for the development of PPS after ESD specifically include: female sex, sessile lesions and resection time > 90 min [5, 7].

To distinguish PPS from colonic perforation can be challenging and critical since follow-up and prognosis are abysmally different. PPS has a favorable prognosis: is managed without surgery, in contrast to perforation, which has high short-term morbimortality and requires urgent surgical management [9]. The gold standard for the diagnosis is abdominal CT, in which the absence of extra-luminal air rules out the presence of intestinal perforation. CT can also show severe mural thickening with a stratified enhancement pattern, a mural defect filled with fluid and surrounding infiltration without extra-luminal air [10]. Abdominal X-ray has a low sensitivity to identify pneumoperitoneum (30–59%) and does not allow to identify the site of a perforation [10].

Treatment of PPS is conservative: bowel rest, parenteral fluids and broad-spectrum antibiotics. Patients with mild symptoms may have an early discharge on a liquid diet for 1–2 days and oral antibiotics [4, 11]. Severe symptoms imply a longer in-hospital stay for observation, parenteral analgesics and antibiotics.

Theoretically, submucosal injection of saline solution into sessile injuries prior to electrocautery resection, like the one done in this case, may reduce the risk of PPS; however, there are not comparative studies in actual literature that support this practice [4]. Another method to try to prevent this outcome, was recently described by Yamasaki *et al*. They proposed a complete closure of the defect by endoscopy with assisted linear clip (LACC). It showed to have lower postoperative complications; nonetheless, large scale studies are required to have greater strength and quality of evidence [12].

## CONCLUSION

PPS is a rare complication, getting to share cases like this one can allow physicians to considered PPS as a possible complication in the context of patients who have undergone endoscopic resections with electrocoagulation. Recognizing the clinical and imaging patterns related to PPS allow physicians to carry out a proper approach. More studies needed to create preventive measures to avoid PPS.

## CONFLICT OF INTEREST STATEMENT

None declared.

## FUNDING

Funding for publishing open access was provided by Hospital Universitario Mayor Mederi. No funding was required for article writing and editing.

## References

[1] Cha J , Lim K, Lee S, Joo Y, Hong S, Kim T, et al. Clinical outcomes and risk factors of post-polypectomy coagulation syndrome: a multicenter, retrospective, case-control study. Endoscopy 2013;45:202–7. 23381948 10.1055/s-0032-1326104

[2] Nakajima T , Saito Y, Tanaka S, Iishi H, Kudo S, Ikematsu H, et al. Current status of endoscopic resection strategy for large, early colorectal neoplasia in Japan. Surg Endosc 2013;27:3262–70. 23508817 10.1007/s00464-013-2903-x

[3] Yamashina T , Takeuchi Y, Uedo N, Hamada K, Aoi K, Yamasaki Y, et al. Features of electrocoagulation syndrome after endoscopic submucosal dissection for colorectal neoplasm. J Gastroenterol Hepatol 2016;31:615–20. 26202127 10.1111/jgh.13052

[4] Hirasawa K . Coagulation syndrome: delayed perforation after colorectal endoscopic treatments. World J Gastrointestinal Endosc 2015;7:1055. 10.4253/wjge.v7.i12.1055 PMC4564832 26380051

[5] Arimoto J , Higurashi T, Kato S, Fuyuki A, Ohkubo H, Nonaka T, et al. Risk factors for post-colorectal endoscopic submucosal dissection (ESD) coagulation syndrome: a multicenter, prospective, observational study. Endosc Int Open 2018;06:E342–9. 10.1055/s-0044-101451 PMC5842075 29527556

[6] Nakajima T , Saito Y, Tanaka S, Iishi H, Kudo S, Ikematsu H, et al. Current status of endoscopic resection strategy for large, early colorectal neoplasia in Japan. Surg Endosc 2013;27:3262–70. 23508817 10.1007/s00464-013-2903-x

[7] Ito S , Hotta K, Imai K, Yamaguchi Y, Kishida Y, Takizawa K, et al. Risk factors of post-endoscopic submucosal dissection electrocoagulation syndrome for colorectal neoplasm. J Gastroenterol Hepatol 2018;33:2001–6. 29864790 10.1111/jgh.14302

[8] Sagawa T . Analysis of colonoscopic perforations at a local clinic and a tertiary hospital. World J Gastroenterol 2012;18:4898. 23002362 10.3748/wjg.v18.i35.4898 PMC3447272

[9] Kim HW . What is different between postpolypectomy fever and postpolypectomy coagulation syndrome? Clin Endosc 2014;47:205. 24944980 10.5946/ce.2014.47.3.205 PMC4058534

[10] Shin YJ , Kim YH, Lee KH, Lee YJ, Park JH. CT findings of post-polypectomy coagulation syndrome and colonic perforation in patients who underwent colonoscopic polypectomy. Clin Radiol 2016;71:1030–6. 27085213 10.1016/j.crad.2016.03.010

[11] Kim ER , Chang DK. Management of complications of colorectal submucosal dissection. Clin Endosc 2019;52:114–9. 30959586 10.5946/ce.2019.063 PMC6453857

[12] Yamasaki Y , Takeuchi Y, Iwatsubo T, Kato M, Hamada K, Tonai Y, et al. Line-assisted complete closure for a large mucosal defect after colorectal endoscopic submucosal dissection decreased post-electrocoagulation syndrome. Dig Endosc 2018;30:633–41. 29573468 10.1111/den.13052

